# First record of Acaenitinae (Hymenoptera, Ichneumonidae) from South America with description of a new species and a key to the world species of *Arotes* Gravenhorst

**DOI:** 10.3897/zookeys.137.1788

**Published:** 2011-10-14

**Authors:** Carol Castillo, Ilari E. Sääksjärvi, Andrew M.R. Bennett, Gavin R. Broad

**Affiliations:** 1 Zoological Museum, Section of Biodiversity and Environmental Sciences, Department of Biology, FIN-20014, University of Turku, Finland; 2 Canadian National Collection of Insects, Agriculture and Agri-Food Canada, Ottawa, Ontario K1A 0C6, Canada; 3 Department of Entomology, Natural History Museum, Cromwell Road, London, London SW7 5BD, UK

**Keywords:** Amazonia, *Arotes*, Neotropics, parasitoid wasp, Peru, rainforest

## Abstract

A new species of Acaenitinae, *Arotes ucumari* Castillo & Sääksjärvi, **sp. n.**, is described and illustrated representing the first record of the subfamily from South America. The new species was collected from a premontane tropical rain forest in the Peruvian Andes at 1500 m. A key to the world species of *Arotes* Gravenhorst,1829 is provided. The subspecies *Arotes albicinctus moiwanus* (Matsumura, 1912)is raised to species rank, *Arotes moiwanus* **stat. n.**

## Introduction

The Acaenitinae is one of the most conspicuous subfamilies of Ichneumonidae (Hymenoptera). It is clearly monophyletic as defined by at least one striking synapomorphy: the very long and triangular hypopygium of the female, although less developed in some species of the *Coleocentrus* group. The subfamily was traditionally classified in two tribes, the Acaenitini and Coleocentrini (Townes 1971). However, Wahl and Gauld (1998) suggested that this classification be discontinued as Coleocentrini is paraphyletic with regard to Acaenitini.

Many acaenitines are large in size, vividly coloured and possess long ovipositors. Despite this, little is known about their biology and specimens are rare in entomological collections. Many species of the subfamily live in ancient forests and in the Neotropics have been found in highlands (Gauld 1991). Most genera occur in the Old World, whereas only five have been reported from the New World (Townes 1971; Gauld 1984, 1991). Only one of these, *Arotes* Gravenhorst, has previously been reported from the Neotropics, where two species, *Arotes pammae* Gauld and *Arotes facialis* (Cameron) have been described from Costa Rica and Guatemala respectively (Cameron 1886; Gauld 1991). In addition, *Coleocentrus rufus* Provancher has been collected from the Cayman Islands (a single specimen in CNC). Townes and Townes (1960) suggested an Old World origin for the subfamily with a relatively recent conquest of the New World via the Bering Strait.

The aim of the present paper is to describe a new species of *Arotes* from the tropical Peruvian Andes. Also, we raise to species rank *Arotes moiwanus* stat. n. and provide a key to the world species of the genus.

## Material and methods

The only known specimen of the new species is deposited in The Natural History Museum, University of San Marcos, Lima, Peru (UNSM). The specimen is currently on loan to the Zoological Museum, University of Turku, Finland (ZMUT). We searched for more Neotropical specimens of *Arotes* at the Natural History Museum, London (NHM), the Canadian National Collection of Insects, Ottawa (CNC) and the American Entomological Institute, Gainesville, Florida (AEI).

To verify the new species status of our Peruvian specimen, we examined specimens of 10 of the 15 previously described *Arotes* species in CNC and NHM and also compared it to descriptions of all the described species. We were not able to examine specimens of *Arotes annulicornis* Kriechbaumer, *Arotes flaviscutatus* Wang & Huang, *Arotes odontus* Uchida, *Arotes nigricoxis* (Förster) and *Arotes sugiharai* Uchida. All species except for *Arotes nigricoxis* have been included in the key on the basis of original descriptions, online images of Japanese specimens (Hokkaido University, http://neosci-gw.museum.hokudai.ac.jp/html/modules/pukiwiki/641.html), and their inclusion in previous keys, namely those of Uchida (1934), Kolarov (1997) and Sheng and Sun (2009). The presented key is modified from the keys of Townes and Townes (1960), Gauld (1991) and Kolarov (1997).

Observations were made using Olympus SZX10 and SZ40 stereomicroscopes. Layer photos of the holotype were taken using an Olympus SZX16 with motorized focus drive attached to an Olympus E520 digital camera. Digital photos were combined by using the programmes Deep Focus 3.1 and Quick PHOTO CAMERA 2.3. Images of specimens in BMNH were taken with a Canon EOS 450D digital camera attached to a Leica MZ12 stereomicroscope and with a Canon EOS 450D with a Pentax 50 mm macro lens. Several partially focused images were combined using Helicon Focus v. 4.80 software. Digital photos at CNC were made using a Leica MZ16 stereomicroscope with motorized focus drive attached to a Leica DFC420 digital camera. Photos were combined using Leica Application Suites Montage Multifocus software. Morphological terminology and forms of description follow those of Gauld (1991).

## Taxonomy

### Genus Arotes

Gravenhorst

http://species-id.net/wiki/Arotes

Arotes Gravenhorst, 1829: 449. Type: *Arotes albicinctus* Gravenhorst, by monotypy.
Asthenomeris Förster, 1869: 168. Type: *Asthenomeris nigricoxis* Förster, 1888, by subsequent inclusion by Schmiedeknecht 1888. Synonymized by Townes et al. 1965.
Sphalerus Kriechbaumer, 1878: 41. Type: *Sphalerus bifasciatus* Kriechbaumer (= *albicinctus*), by monotypy.
Retanisia Cameron, 1886: 299. Type: *Retanisia facialis* Cameron, by monotypy. Synonymized by Townes and Townes 1960.

#### Diagnosis.

*Arotes* can be distinguished from other genera of Acaenitinae by combination of both of the following characters: hind tarsal claws with a sharp, accessory tooth near apex of claw and areolet of fore wing open with intercubitus distal to vein 2*m-cu*.

##### Description.

Moderately large wasps, mostly black, black and white or black and yellow; legs may be reddish or yellowish in part; antennae with or without white band; fore wing with or without dark spots. Mandible with dorsal tooth equal to or slightly shorter than ventral tooth; clypeus with a pre-apical transverse ridge, the apical edge with medial tubercle in most species; subocular sulcus complete; face centrally swollen, with weak transverse ridges or weak central rugose ridges, and with median vertical ridge which extends between antennae and onto frons as distinct carina; occipital carina complete dorsally. Notaulus strong, reaching posteriorly to centre of mesoscutum; scutellum flattened, laterally carinate at least at anterior end; submetapleural carinae more or less complete, not expanded anteriorly; propodeum quite long, with more or less clearly defined area superomedia; propodeal spiracle elliptical. Fore wing without areolet, intercubitus distal to 2*m-cu*; vein 2*m-cu* with two well-separated bullae. All tarsal claws with sharp, accessory tooth near apex of claw. Ventral swelling of 1^st^ sternite from acute to smoothly rounded, bearing numerous, long, erect hairs; ovipositor projecting beyond apex of metasoma by about 1.8-2.7 times length of hind tibia; ovipositor with ventral ridges apically that are vertical and widely spaced.

##### Key to the world species of *Arotes*

**Note.** *Asthenomeris nigricoxis* Förster is a species of *Arotes* according to Townes et al. (1965), but specimens of this species are not known to us and the original description (Schmiedeknecht 1888) is too brief to allow diagnosis from its congeners. For this reason it is omitted from the key below.

Specimens at CNC with intermediate colour patterns indicate that *Arotes albicinctus* (Gravenhorst) and *Arotes annulicornis* Kriechbaumer may be synonyms.

**Table:** 

1	Propodeum completely black (Fig. 1)	2
–	Propodeum with at least some light colour (Figs 2-3)	8
2(1)	Fore wing with one or two discrete, dark spots (Figs 4-5) or, if male and spot indistinct, antennal flagellum apically broadly yellow-white	3
–	Fore wing lacking discrete, dark spots, at most vaguely infuscate on apical margin; male antenna not broadly yellow-white apically	7
3(2)	Fore wing with two discrete dark marks, one at apex of wing and one adjacent to pterostigma	*Arotes sugiharai* Uchida (eastern Palaearctic: Japan)
–	Fore wing with only apical dark mark	4
4(3)	Flagellum entirely dark	*Arotes facialis* (Cameron) (Neotropical: Guatemala)
–	Flagellum not entirely dark: lighter ventrally than dorsally and/ or with a medial light band	5
5(4)	Hind femurpredominantly orange-red	*Arotes ustulatus* Kriechbaumer (western Palaearctic)
–	Hind femur predominantly black or dark brown	6
6(5)	Hind tibia orange to orange-brown in basal half	*Arotes odontus* Uchida (eastern Palaearctic: Russia – Sakhalin Oblast)
–	Hind tibia pale yellow or ivory in basal half	*Arotes maurus* Rohwer (some specimens) (western and central Nearctic)
7(2)	First and second tergites with light-coloured posterior margins	*Arotes albicinctus* (Gravenhorst) (trans-Palaearctic and Oriental)
–	First and second tergites completely black	*Arotes annulicornis* Kriechbaumer (western and central Palaearctic)
8(1)	Hind tibia entirely black except may be narrowly light coloured at extreme base	9
–	Hind tibia broadly yellow/white at base, can be black apically, or mostly yellow or red	10
9(8)	Antennal flagellum without white band (Fig. 4); first sternite sub-basally with smoothly rounded projection	*Arotes ucumari* sp. n. (Neotropical: Peru)
–	Antennal flagellum with white band (Fig. 5); first sternite sub-basally with acutely angled, sharp projection	*Arotes pammae* Gauld (Neotropical: Costa Rica)
10(8)	Hind tibia completely yellow or orange, at most, slightly darker orange at apex	11
–	Hind tibia with some dark colour (black or brown)	13
11(10)	First sternite sub-basally strongly convex, like a tubercle	*Arotes flaviscutatus* Wang & Huang (Oriental: China – Fujian Province)
–	First sternite sub-basally weakly convex, not tuberculate	12
12(11)	Mesoscutum black or orange, sometimes with restricted lighter coloured areas, but not a continuous lighter coloured stripe along notaulus; middle of pronotum just dorsoposterior to pronotal trough almost impunctate, punctures separated by much more than their diameter	*Arotes melleus* (Say) (some specimens) (central and eastern Nearctic)
–	Mesoscutum black with extensive yellow or white regions, notaulus completely encompassed by a wide yellow or white stripe; middle of pronotum punctate, punctures separated by their own diameter or less	*Arotes decorus* Say (southcentral and eastern Nearctic)
13(10)	Mesoscutum black or orange, notaulus not completely encompassed by a wide yellow or white stripe	14
–	Mesoscutum black with extensive yellow or white regions, notaulus completely encompassed by a wide yellow or white stripe	16
14(13)	Metapleuron extensively rugose to rugoso-punctate (Fig. 6)	*Arotes maurus* Rohwer (some specimens) (western and central Nearctic)
–	Metapleuron finely to densely punctate without rugosity	15
15(14)	Metapleuron polished with fine punctures separated by much more than their diameter (Fig. 7); hind femur ventrally evenly narrowing subapically towards apex; female with mesoscutum ranging from completely fulvous (most specimens) to completely black	*Arotes melleus* (Say) (some specimens) (central and eastern Nearctic)
-	Metapleuron sub-polished with coarser punctures separated by their own diameter or less; hind femur ventrally with a strong, subapical swelling that narrows abruptly towards apex; female with mesoscutum completely black	*Arotes moiwanus* (Matsumura) (eastern Palaearctic and Oriental)
16(13)	Hind tibia with at least basal 0.4 light coloured (basal 0.6 light in some specimens)	*Arotes amoenus* Cresson (southcentral and eastern Nearctic)
–	Hind tibia with no more than basal 0.2 light coloured	*Arotes maculatus* Sheng & Sun (eastern Palaearctic: China – Henan Province)

### Arotes ucumari

Castillo & Sääksjärvi sp. n.

urn:lsid:zoobank.org:act:6F98E674-ED64-4EEA-9DD9-3DAD72D6C30F

http://species-id.net/wiki/Arotes_ucumari

#### Type locality.

Peru, Dept. of Cusco, Manu National Park, Cosñipata valley, San Pedro, 13°02'58"S, 71°32'13"W, 1500 m elev., C. Castillo leg., 20 September 2007.

#### Type specimen.

 Holotype female, pinned. Original label: “Peru, CU, San Pedro, 13°02'58"S, 71°32'13"W, 1500 m, Malaise trap, 20.ix.2007, C. Castillo". UNSM.

#### Diagnosis.

*Arotes ucumari* sp. n. (Fig. 4) can be distinguished from all other described *Arotes* spp. by combination of all the following characters: 1) hind tibia black; 2) scutellum yellow; 3) antenna without a medial light coloured band; 4) hind femur ventrally not or only slightly swollen subapically.

**Figures 1–3.**
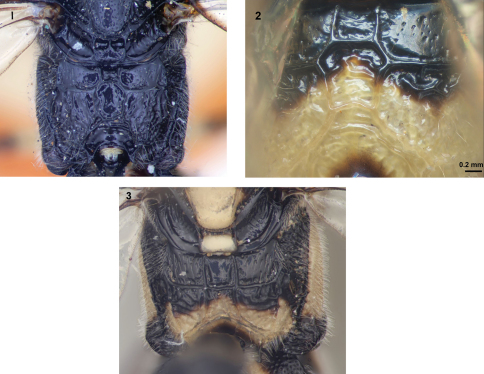
Propodeum of neotropical species of *Arotes*, posterodorsal view. **1**. *Arotes facialis*, **2**. *Arotes ucumari* sp. n., **3**. *Arotes pammae*.

*Arotes ucumari* sp. n. is readily distinguished from other New World species of *Arotes* (except *Arotes facialis*) on account of its totally black antennae (character 3). It differs from *Arotes facialis* in that it has more extensive yellow colouration on the meso- and metasoma (*Arotes facialis* is almost completely black). In addition, the propodeal carination of the three neotropical species is different (Figs 1-3). The area superomedia of *Arotes facialis* is hexagonal to subcircular whereas that of *Arotes ucumari* is irregularly octagonal (Fig. 2). In coloration, *Arotes ucumari* is similar to *Arotes pammae* Gauld (Fig. 5) but may be separated from that species by the black antennae, smoothly rounded first sternite of metasoma and the propodeal carination (in *Arotes ucumari*, the anterior transverse carina joins the area superomedia at its upper half and the shape of the area superomedia is irregularly octagonal).

#### Description.

##### Female.

**Figures 4–5.**
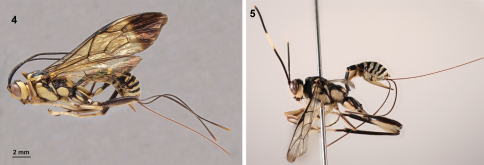
Habitus, lateral view. **4**. *Arotes ucumari* sp. n., holotype female (Peru). **5**. *Arotes pammae*.

Habitus in Figure 4. Lower face broad, inner margins of eyes ventrally divergent; frons concave, smooth; antenna with 34 flagellomeres; antenna about as long as fore wing. Pronotum with striae directed to hind corner of pronotum, middle of pronotum just dorsoposterior to pronotal trough impunctate; mesoscutum with lobes sparsely, coarsely punctate, closely punctate on front side; scutellum more closely punctate than mesoscutum; mesopleurum anteriorly, ventrally coarsely punctate; metapleurum coarsely, closely punctate but, above submetapleural carina punctures are separated by more than their diameter; propodeum with area superomedia clearly delineated, almost hexagonal anteriorly, posteriorly narrowed, so that it is irregularly octagonal (Fig. 2), posterior border of area superomedia concave; anterior transverse carina joining area superomedia at its upper half; lateral longitudinal carina only delineating area externa and area posteroexterna; area petiolaris confluent with area posteroexterna. Fore wing length 14 mm, wing without areolet, with cross vein 2*rs-m* (or 3*rs-m*, depending on interpretation) distal to 2*m-cu*. Hind femur ventrally with a slight subapical swelling. First metasomal sternite with projection smoothly rounded; ovipositor projecting beyond apex of metasoma by about 1.9 times length of hind tibia.

Yellowish species with black marks. Head light yellow with temple, frons and inner margin of occiput black; antenna black except infuscate tip on last flagellomere. Mesosoma mostly light yellow with dorsal and hind margins of pronotum black, mesoscutum black with yellow marks on lateral and hind regions of central lobe, U-shaped mark in dorsal view, lateral sides of mesoscutum, scutellum and metanotum also yellow, hind margin of mesopleurum, mesosternum and anterior half of propodeum black. Wings slightly yellowish, with apex broadly infumate, pterostigma and veins black. Fore and mid legs with light yellow on dorsal surfaces of trochanters and femora, most of tibiae and all tarsi infuscate; hind leg black with yellow marks on lower half of coxa, two oval yellow marks on dorsal and lateral sides of coxa, most of trochanter and ventral half of femur light yellow. Metasoma black, tergites 1-2 with broad yellowish marks close to hind margin, tergite 3 almost entirely black, tergites 4+ with hind margins and lateral spots light yellow; subgenital plate infuscate with upper margin yellowish; ovipositor orange, ovipositor sheaths black with dull yellow tip.

##### Male.

Unknown.

##### Biology.

The host of *Arotes ucumari* sp. n. is not known. North American *Arotes* species have been reared from *Melandrya* (Melandryidae), *Leptura* (Cerambycidae) and *Tomoxia* (Mordellidae) (Townes and Townes 1960; Gauld 1991).

##### Ecology.

The type locality is in a primary forest at the south east limit of Manu National Park. On the eastern slopes of the Andes, this altitude (1500 m) is considered as a major ecotone between the humid montane forest and the premontane forest belt (Young and León 1999). It differs from both highland Andean and lowland Amazonian vegetation formations. The Andean foothills of Manu-Tambopata are considered to be a super-humid region (Killeen 2007). The annual precipitation in 2007, when the holotype was collected, was 3158 mm. The mean maximum and minimum temperatures were between 21,6 and 11,3 degrees Celsius (SENAMHI, National Service of Meteorology and Hydrology of Peru).

##### Etymology.

Ucumari is the quechuan name for the only South American species of bear, *Tremarctos ornatus*, the Spectacled Bear. Just as is possible in the case of the Acaenitinae parasitoid wasps, the tremarctine bears reached the New World via the Bering Strait, and expanded their range southwards into North and South America. By naming the new species as *Arotes ucumari* we hope to draw attention to the conservation of both of these rare tropical Andean species.

##### The status of *Phaenolobus* (*Acoenitus*) *moiwanus* Matsumura, 1912

During the process of comparing specimens to verify the new species status of *Arotes ucumari*, examination of material of *Arotes albicinctus* and its subspecies *Arotes albicinctus moiwanus* at NHM and CNC revealed differences that could indicate that these two forms may represent two distinct species. Since Uchida (1934), *Phaenolobus* (*Acoenitus*) *moiwanus* Matsumura, 1912 has been regarded as a subspecies of *Arotes albicinctus* (Gravenhorst, 1829) (e.g. Townes et al. 1965; Yu and Horstmann 1997). Our examination found the differences listed in Table 1.

In most ichneumonid species in which subspecies are recognized, the only indicator of subspecies is colour, not sculpture. For example, *Campoplex sugiharai sugiharai* (Uchida), *Campoplex sugiharai australis* Momoi and *Campoplex sugiharai okinawensis* Momoi (see Momoi 1970). Such a major difference in the sculpture of the frons (striate versus sparsely punctate) generally indicates two species. We believe that the sculptural differences of the frons correlated with major colour differences of the scutellum and propodeum are clear indicators that *Arotes albicinctus* and *Arotes moiwanus* are two distinct species. *Arotes moiwanus* stat. n. is hereby recognized as a valid species. We have not seen males of *Arotes moiwanus* but we expect these characters to remain valid.

**Figures 6–7.**
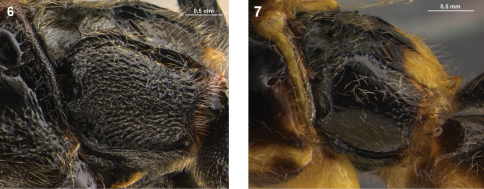
Metapleuron, lateral view. **6**. *Arotes maurus*, **7**. *Arotes melleus*.

## Discussion

Whereas description of a species based on a single specimen is not ideal, we are confident that the species is distinct based on the unique combination of characters listed in the diagnosis and our assessment of species-specific characters within the genus *Arotes*.  For example, the presence of a completely black flagellum in *Arotes ucumari* is very rare for the genus (in the previously described species only *Arotes facialis *from Guatemala has this character, all other species have either a medial pale band or are lighter on the ventral surface of the flagellum than the dorsal surface). It is highly unlikely that *Arotes ucumari* and *Arotes facialis* are conspecific because the body of *Arotes facialis* is almost completely black (Gauld 1991), whereas *Arotes ucumari* has large yellow regions on the body (Fig. 4).  In addition, the area superomedia of *Arotes facialis* is hexagonal to subcircular (Fig. 1) compared to *Arotes ucumari* in which this area is much wider anteriorly than posteriorly (Fig. 2).

In our opinion, it is important to describe the new species now, rather than wait for additional material to be collected. Considering the extensive collecting done in this region, for example, the Colombian Arthropod Project (CAP) from 2001 to 2003, 188 Malaise trap months in Peru from 1998-2001 (Sääksjärvi et al. 2004) and 6 years of canopy fogging in Ecuador since 1994 (Erwin et al. 2005), it is unlikely that a great number of additional acaenitines will be rapidly collected.  The description of this species will draw attention to the presence of acaenitines in South America which will hopefully lead to the discovery of additional material (both in collections and from future collecting by other individuals).

**Table 1.** Key morphological differences between *Arotes albicinctus* and *Arotes moiwanus*

*Arotes albicinctus*	*Arotes moiwanus*
Frons with strong oblique striations extending from medial ocellus towards eye. Orbit with dense, coarse punctures near antenna (Fig. 8).	Frons with weak striations or striations absent between medial ocellus and eye. Orbit with moderately fine, sparse punctures near antenna (Fig. 9).
Scutellum and propodeum black.	Scutellum and propodeum marked with light colour (creamy-white to yellow).

**Figures 8–9.**
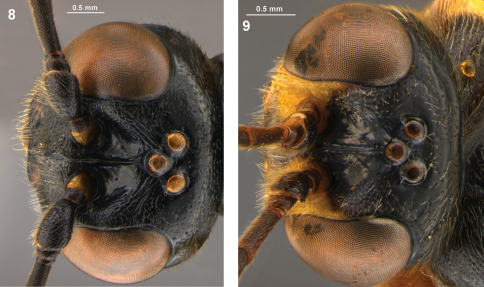
Head, dorsal view. **8**. *Arotes albicinctus*, **9**. *Arotes moiwanus*.

## Supplementary Material

XML Treatment for Arotes

XML Treatment for Arotes ucumari

## References

[B1] Cameron P (1886) Insecta. Hymenoptera (Families Tenthredinidae-Chrysididae). Biologia Central-Americana 1: 299-300.

[B2] Erwin TL Pimienta MC Murillo OE Ascherov (2005) Mapping Patterns of Diversity for Beetles Across the Western Amazon Basin: A Preliminary Case for Improving Inventory Methods and Conservation Strategies. Proceedings of the California Academy of Sciences 56: 72-85.

[B3] Förster A (1869) Synopsis der Familien und Gattungen der Ichneumonen. Verhandlungen des Naturhistorischen Vereins der Preussischen Rheinlande und Westfalens 25 (1868): 135-221.

[B4] Gauld ID (1984) The Pimplinae, Xoridinae, Acaenitinae and Lycorininae (Hymenoptera: Ichneumonidae) of Australia. Bulletin of the British Museum (Natural History) 49 (4): 1-339.

[B5] Gauld ID (1991) The Ichneumonidae of Costa Rica, 1. Memoirs of the American Entomological Institute 47: 1-589.

[B6] Gravenhorst JLC (1829) Ichneumonologia Europaea, Vratislaviae 3, 1097 pp.

[B7] Hokkaido University (2011)Hokkaido University, Collection of Systematic Entomology. http://neosci-gw.museum.hokudai.ac.jp/html/modules/pukiwiki/641.html [accessed 7 September 2011]

[B8] Killeen TJ Douglas M Consiglio T Jørgensen PM Mejia J (2007) Dry spots and wet spots in the Andean hotspot. Journal of Biogeography 34: 1357-1373. 10.1111/j.1365-2699.2006.01682.x

[B9] Kolarov J (1997) Hymenoptera, Ichneumonidae. Part I. Pimplinae, Xoridinae, Acaenitinae, Collyriinae. Fauna Bulgarica 25: 1-322.

[B10] Kriechbaumer J (1878) Neue Schlupfwespen aus Ungarn. Entomologische Nachrichten 4 (4): 41-46.

[B11] Matsumura S (1912) Thousand insects of Japan. Supplement IV. Tokyo, 247 pp.

[B12] Momoi S (1970) Ichneumonidae (Hymenoptera) of the Ryukyu Archipelago. Pacific Insects 12: 327-399.

[B13] Sääksjärvi IE Haataja S Neuvonen S Gauld ID Jussila R Salo J Burgos AM (2004) High local species richness of parasitic wasps (Hymenoptera: Ichneumonidae; Pimplinae and Rhyssinae) from the lowland rainforests of Peruvian Amazonia. Ecological Entomology 29: 735-743. 10.1111/j.0307-6946.2004.00656.x

[B14] Schmiedeknecht O (1888) Die europaischen Gattungen der Schlupfwespen Familie Pimplariae. Zoologische Jahrbucher Abteilung fur Systematik 3: 419-444.

[B15] SENAMHI (2010) SENAMHI National Service of Meteorology and Hydrology of Peru. Data obtained by request on April 2010. http://www.senamhi.gob.pe/

[B16] Sheng ML Sun SP (2009) Insect fauna of Henan, Hymenoptera: Ichneumonidae. Science Press, Beijing, 340 pp.

[B17] Townes H (1971) The Genera of Ichneumonidae, part 4. Memoirs of the American Entomological Institute 17: 1-372.

[B18] Townes H Townes M (1960) Ichneumon-Flies of America North of Mexico: 2. Subfamilies Ephialtinae, Xoridinae, Acaenitinae. United States National Museum Bulletin 216 (2): 1-676. doi: 10.5479/si.03629236.216.1-2

[B19] Townes HK Momoi S Townes M (1965) A catalogue and reclassification of the eastern Palearctic Ichneumonidae. Memoirs of the American Entomological Institute 5: 1-661.

[B20] Young K León B (1999) Peru’s humid eastern montane forests: an overview of their physical settings, biological diversity, human use and settlement, and conservation needs. DIVA, Technical Report no 5.

[B21] Yu DS Hortsmann KA (1997) A catalogue of world Ichneumonidae (Hymenoptera). Parts I-II. Memoirs of the American Entomological Institute 58: 1-1558.

[B22] Uchida T (1928) Dritter Beitrag zur Ichneumoniden-Fauna Japans. Journal of the Faculty of Agriculture, Hokkaido University 25: 1-115.

[B23] Uchida T (1934) Beiträge zur Systematik der Tribus Acoenitini Japans (Hym. Ichneum. Pimplinae). Insecta Matsumurana 9: 41-54.

[B24] Wahl DB Gauld ID (1998) The cladistics and higher classification of the Pimpliformes (Hymenoptera: Ichneumonidae). Systematic Entomology 23: 265-298. 10.1046/j.1365-3113.1998.00057.x

